# Raman-Enhanced Phase-Sensitive Fibre Optical Parametric Amplifier

**DOI:** 10.1038/srep20180

**Published:** 2016-02-02

**Authors:** Xuelei Fu, Xiaojie Guo, Chester Shu

**Affiliations:** 1 Department of Electronic Engineering and Center for Advanced Research in Photonics, The Chinese University of Hong Kong, Shatin, N.T., Hong Kong

## Abstract

Phase-sensitive amplification is of great research interest owing to its potential in noiseless amplification. One key feature in a phase-sensitive amplifier is the gain extinction ratio defined as the ratio of the maximum to the minimum gains. It quantifies the capability of the amplifier in performing low-noise amplification for high phase-sensitive gain. Considering a phase-sensitive fibre optical parametric amplifier for linear amplification, the gain extinction ratio increases with the phase-insensitive parametric gain achieved from the same pump. In this work, we use backward Raman amplification to increase the phase-insensitive parametric gain, which in turn improves the phase-sensitive operation. Using a 955 mW Raman pump, the gain extinction ratio is increased by 9.2 dB. The improvement in the maximum phase-sensitive gain is 18.7 dB. This scheme can significantly boost the performance of phase-sensitive amplification in a spectral range where the parametric pump is not sufficiently strong but broadband Raman amplification is available.

Phase-sensitive amplification (PSA) has been investigated extensively in recent years in view of its potential to achieve noiseless amplification and non-classical squeezed states generation^1^. Relying on the nonlinear interactions, PSA can take place in both *χ*^(2)^ materials such as periodically-poled lithium niobate waveguides^2^ and *χ*^(3)^ materials such as highly nonlinear fibres^3^,^4^ (HNLFs) and dispersion engineered nonlinear waveguides^5^,^6^. Due to its ability to selectively amplify input optical signals at certain phases, PSA has been considered for various applications in optical communications, including low-noise amplification^7^, regeneration^4^, format conversion^8^ of phase-encoded signals, as well as optical signal-to-noise ratio (OSNR) improvement^9^.

Low-loss HNLF allows the use of a long interaction length for nonlinear amplification of signals. Combined with the mature development of dispersion engineering, HNLF serves as a desired platform for the investigation of PSA. HNLF based PSA has been demonstrated based on two schemes: nonlinear optical loop mirror^10^,^11^ and phase-sensitive (PS) fibre optical parametric amplifier (FOPA)^1^,^4^,^7^,^8^,^9^. In the nonlinear optical loop mirror based PSA, the pump and signal are located at the same wavelength. As a result, guided acoustic-wave Brillouin scattering^12^ of the pump directly affects the signal quality at low frequencies^11^. Hence, recent research efforts have been focused mostly on PS-FOPA based PSA.

PS-FOPA can be realized either in a frequency degenerate manner^4^, in which the signal and idler are located at the same wavelength, or in a frequency non-degenerate manner^1^, in which the signal and idler have different wavelengths. The frequency non-degenerate PS-FOPA is format transparent and can potentially support multi-wavelength operation^1^,^7^. In the following demonstration, we thus adopt the frequency non-degenerate PS-FOPA encompassing a single pump, a signal, and an idler at the input.

As opposed to the phase-insensitive (PI) amplifiers, the performance of a PSA is not only quantified by the signal gain, but also the gain extinction ratio (GER). The GER describes the ability of the PSA in amplifying/deamplifying input signals at selective phases. In PSA based low-noise amplification, a large GER combined with a high PS gain implies a small noise figure^1^. Considering a PS-FOPA in the linear amplification regime, the GER increases with the PI parametric gain attainable from the same parametric pump in PI amplification.

The introduction of a backward Raman pump to a conventional PI-FOPA has proved to substantially increase the signal gain^13^. The enhancement is contributed by both the direct Raman gain and the increased PI parametric gain from the Raman-amplified parametric pump. Recent investigations also reveal the capability of the Raman-assisted PI-FOPA in deferring gain saturation^14^ and suppressing multi-channel crosstalk^15^,^16^.

Here, we propose and demonstrate the use of backward Raman amplification to enhance the performance of a conventional PS-FOPA^17^. As a result of increased PI parametric gain from the Raman-amplified parametric pump, the GER of the Raman-enhanced PS-FOPA is increased by 9.2 dB compared to the conventional PS-FOPA. Combined with the direct Raman gain, the maximum PS gain is increased by 18.7 dB.

## Results

### Principle

We first consider a conventional single-pump FOPA which can be modelled by the following matrix^7^


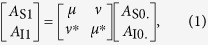


where *A* represents complex amplitude of the optical waves. The first subscripts S and I denote signal and idler, and the second subscripts 0 and 1 denote input and output, respectively. The superscript ∗ denotes conjugation by parametric wavelength conversion. *μ* and *ν* are the complex transfer coefficients of the FOPA. Assuming an undepleted pump, *μ* and *ν* satisfy |*μ*|^2^ − |*ν*|^2^ = 1. Considering PS operation, the output signal power is given by^1^


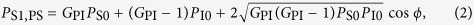


where *P* denotes the power of the optical wave and *G*_PI _= |*μ*|^2^ is the PI parametric gain at the signal wavelength. The PS operation is described through the optical phase *ϕ* =  *ϕ*_*μ*_ + *ϕ*_*υ*_ + *θ*_S0_ + *θ*_I0_ −2*θ*_P0_ when both the signal and idler are present at the amplifier input^1^. Here, *θ*_P0_, *θ*_S0_, and *θ*_I0_ are the input phases of the pump, signal, and idler, and *ϕ*_*μ*_ and *ϕ*_*υ*_ are the phase angles of the complex transfer coefficients *μ* and *ν*, respectively.

**Figure 1:**
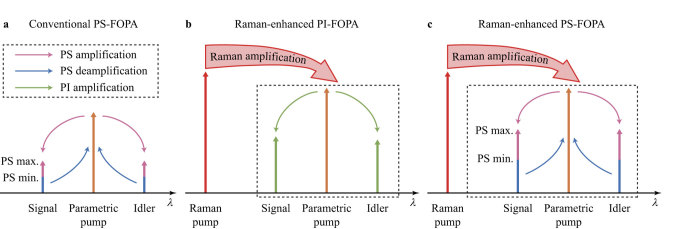
Schematic illustration of conventional and Raman-enhanced fibre optical parametric amplification. (**a**) Conventional phase-sensitive fibre optical parametric amplification (PS-FOPA). (**b**) Raman-enhanced phase insensitive (PI) FOPA with increased signal gain. (**c**) Raman-enhanced PS-FOPA with strengthened PS parametric effect.

Figure 1a illustrates the conventional PS-FOPA process. When *ϕ* = 0, the optical power flows from the parametric pump to the signal and idler at the maximum flow rate, leading to parametric amplification of the signal and idler. On the contrary, the power flows from the signal and idler to the parametric pump when *ϕ* = π, resulting in parametric deamplification. Assuming identical input signal and idler powers, the maximum and minimum PS gains in the conventional PS-FOPA are expressed as


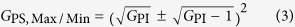


Equation (3) implies that the maximum PS gain increases with *G*_PI_ and approaches 4*G*_PI_, while the minimum PS gain decreases with *G*_PI_ and approaches 0 at large values of *G*_PI_.

The GER, defined as the ratio of the maximum to the minimum PS gains, is thus equal to


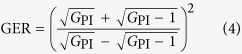


Equation (4) suggests that the GER increases with the PI parametric gain *G*_PI_.

To achieve a higher PI parametric gain, we consider the use of a backward Raman pump to compose a hybrid optical amplifier. Figure 1b illustrates the Raman-enhanced PI-FOPA process. Note that there is no idler present at the amplifier input in this case. The idler shown in Fig. 1b is purely generated from Raman-enhanced PI amplification. Due to direct Raman gain, the parametric pump power increases gradually along the fibre. Consequently, parametric amplification of the signal is strengthened, leading to increased parametric gain. The resulting Raman-amplified PI parametric gain, being larger than *G*_PI,_ is denoted as *G*_RA-PI_. Combined with the direct Raman gain *G*_R_ on the signal, the final PI gain *G*_R_·*G*_RA-PI_ can be substantially larger than *G*_PI_.

Considering PS operation, the amplified parametric pump can enhance either the parametric amplification or deamplification, as schematically illustrated in Fig. 1c. Combined with the direct Raman gain *G*_R_, the Raman-enhanced (RE) maximum and minimum PS gains are given by


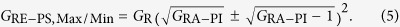


Comparing Equations (5) and (3), it is found that both the direct Raman gain *G*_R_ and the Raman-amplified PI parametric gain *G*_RA-PI_ contribute to the improvement of the maximum PS gain *G*_RE-PS,Max_ over the conventional maximum PS gain *G*_PS,Max_. The influence of the Raman pump on the minimum PS gain deserves more attention. While the direct Raman gain causes an increase in the signal and idler powers, the larger *G*_RA-PI_ leads to a reduction of the minimum PS gain. Hence, the overall increase is smaller than the direct Raman gain alone.

The GER of the Raman-enhanced PS-FOPA is thus given by


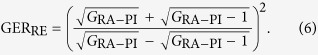


Comparing Equation (6) with (4), we can see that the improvement of GER is contributed solely by 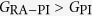.

### Experimental Setup

**Figure 2:**
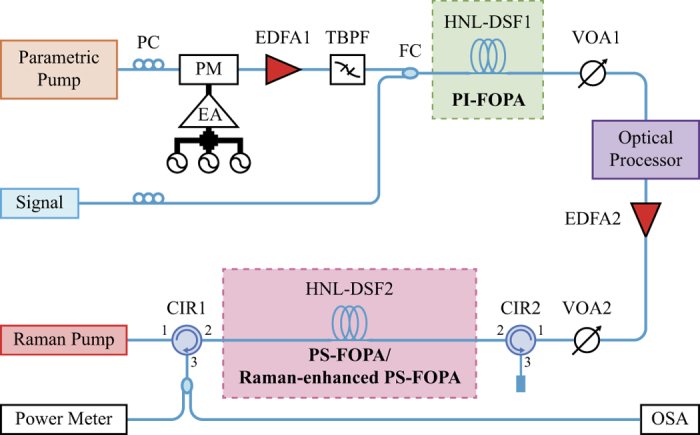
Experimental setup. Arrangement consists of a first PI-FOPA copier stage to generate automatically phase-locked pump, signal, and idler and a second FOPA stage that can switch among the operations of PI-FOPA, PS-FOPA, Raman-enhanced PI-FOPA, and Raman-enhanced PS-FOPA depending on the settings of the optical processor and the Raman pump. PC, polarization controller; PM, phase modulator; EA, electrical amplifier; EDFA, erbium-doped fibre amplifier; TBPF, tunable bandpass filter; FC, fibre coupler; HNL-DSF, highly nonlinear dispersion-shifted fibre; VOA, variable optical attenuator; CIR, optical circulator; OSA, optical spectrum analyser.

Figure 2 shows the experimental setup of the Raman-enhanced PS-FOPA. Phase dithering is applied to the parametric pump to broaden its linewidth so as to suppress stimulated Brillouin scattering (SBS) in the HNLFs. A PI copier stage is adopted to generate a phase-locked three-wave input using automatic phase locking in the PI-FOPA. The copier stage consists of a 2 km highly nonlinear dispersion-shifted fibre (HNL-DSF), which provides 13.9 dB on-off PI parametric gain to the input signal, accompanied by the generation of an idler. The output powers of the signal and idler are nearly the same after the PI copier stage, which is desired in the characterization of the PS-FOPA process. The phases and amplitudes of the pump, signal, and idler are independently adjusted by the optical processor. A 1 km HNL-DSF2 is used for the subsequent demonstration of PS-FOPA and Raman-enhanced PS-FOPA. The Raman pump is introduced through an optical circulator (CIR) to provide backward Raman amplification in HNL-DSF2. The output of the Raman-enhanced PS-FOPA is obtained from Port 3 of CIR1. It is split by a fibre coupler and monitored by a power meter and an optical spectrum analyser (OSA).

### Characterization of Raman-Enhanced PS-FOPA

**Figure 3:**
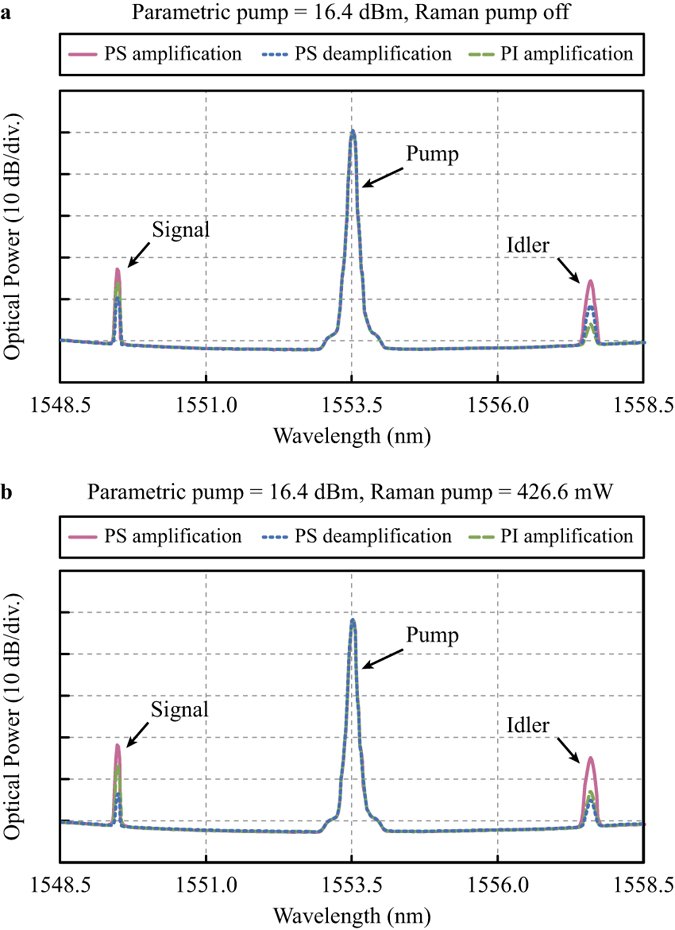
Optical spectra obtained at a parametric pump power of 16.4 dBm. (**a**) Conventional FOPA with no Raman pump. (**b**) Raman-enhanced FOPA with a Raman pump power of 426.6 mW.

**Figure 4:**
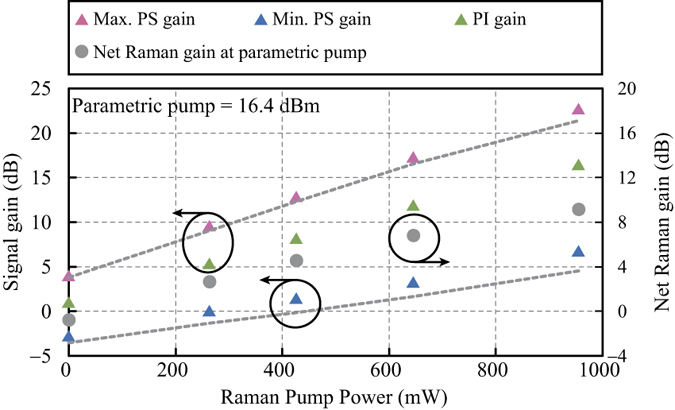
Raman-enhanced PI and PS gains measured at the signal and net Raman gain measured at the parametric pump. The parametric pump power was fixed at 16.4 dBm and the Raman pump power was varied. The maximum and minimum PS gains (pink and blue triangles) are compared with the simulation results (dashed lines).

First, the Raman pump was turned off to realize the conventional single-pump PS-FOPA. Figure 3a shows the output optical spectra at a parametric pump power of 16.4 dBm. The pink and blue curves were obtained at the maximum and minimum PS gains, respectively. The green curve shows the PI-FOPA output and was obtained by blocking the idler with the optical processor such that there was no idler input at HNL-DSF2. With a PI parametric gain of 0.8 dB, the measured maximum and minimum PS gains were 3.9 dB and −2.9 dB, respectively. Note that the insertion loss of the HNLF was excluded from the measurements. The corresponding GER was 6.8 dB. Next, the Raman pump was turned on to 426.6 mW to facilitate Raman-enhanced PS-FOPA. The output optical spectra are plotted in Fig. 3b. Note that the spectral curves are shifted downward by ~7.5 dB with reference to those in Fig. 3a for a better comparison. From the pink and blue curves in Fig. 3b, it is clear that the difference between the maximum and minimum gains has increased. The on-off PI parametric gain was increased by 7.2 dB to 8.0 dB with Raman enhancement. The measured maximum and minimum PS gains were 12.7 dB and 1.3 dB, respectively. Consequently, the corresponding GER was improved by 4.6 dB and attained a value of 11.4 dB. The Raman pump power was then adjusted to different values of 263.0 mW, 645.7 mW, and 955.0 mW for further investigation. The Raman-enhanced PI gain, maximum PS gain, and minimum PS gain were measured. The results were plotted as solid green, pink, and blue triangles respectively in Fig. 4. We also evaluated the maximum and minimum PS gains from numerical calculation. The results were indicated by the dashed grey lines. The numerical model is described in the Method section. The slight discrepancy between the measured and simulated results will be addressed later in this subsection. As a reference, we also plotted in Fig. 4 the net Raman gains experienced by the parametric pump at different Raman pump powers.

**Figure 5:**
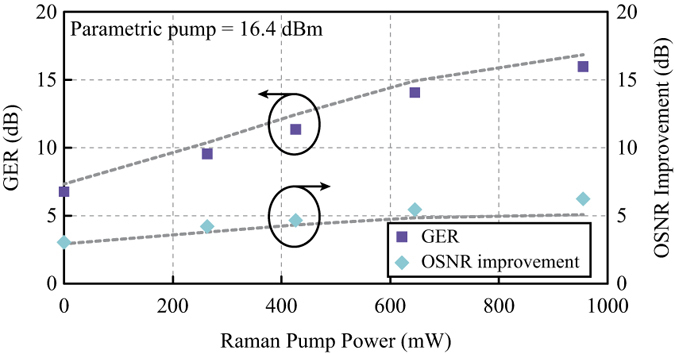
Gain extinction ratio and optical signal-to-noise ratio improvement of Raman-enhanced PS-FOPA. The parametric pump power was fixed at 16.4 dBm and the Raman pump power was varied. The experimentally obtained gain extinction ratio (GER) (squares) and optical signal-to-noise ratio (OSNR) improvement (diamonds) are compared with the simulation results (dashed line).

The dependence of the GER on the Raman pump power was depicted as purple squares in Fig. 5. The dashed grey line was calculated from the numerically derived maximum and minimum PS gains. Compared with the conventional PS-FOPA, a maximum 9.2 dB improvement of the GER was achieved at 955.0 mW Raman pump power. The corresponding maximum PS gain was increased substantially by 18.7 dB.

Comparing the optical spectra of PI and PS amplification in Fig. 3a,b, OSNR improvement of the signal is clearly observed when PS amplification is adopted. The origin of improvement is explained as follows. Since both the signal and idler are loaded-noise limited at the input of HNL-DSF2, incoherent combination of the amplified signal noise and converted idler noise dominates the output noise for both PI-FOPA and PS-FOPA, regardless of whether a Raman pump is used. However, the output signal from the PS-FOPA is the coherent combination of the amplified signal and converted idler, as given by Equation (2). Through coherent combination, extra signal gain is brought by PS amplification. This extra gain can be regarded as an improvement of the output OSNR over the PI-FOPA. The OSNR improvement at different Raman pump powers, indicated by the cyan diamonds, was plotted in Fig. 5. The dashed grey line was obtained from numerical calculation. Judging from Equation (2), the PS gain can be at most 6 dB higher than the PI gain if the signal and idler have the same power. From Fig. 5, it is observed that the OSNR improvement does approach 6 dB as the Raman pump power increases.

**Figure 6:**
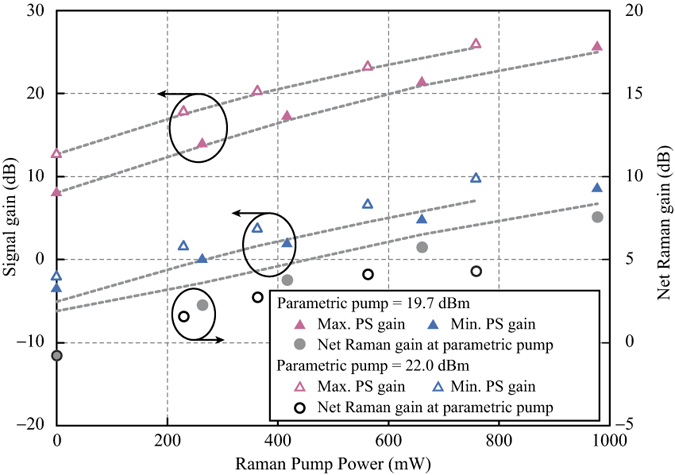
Raman-enhanced PI and PS gains measured at the signal and net Raman gain measured at the parametric pump. The maximum and minimum PS gains (pink and blue triangles) at parametric pump power of 19.7 dBm and 22.0 dBm are compared with the simulation results (dashed lines).

**Figure 7:**
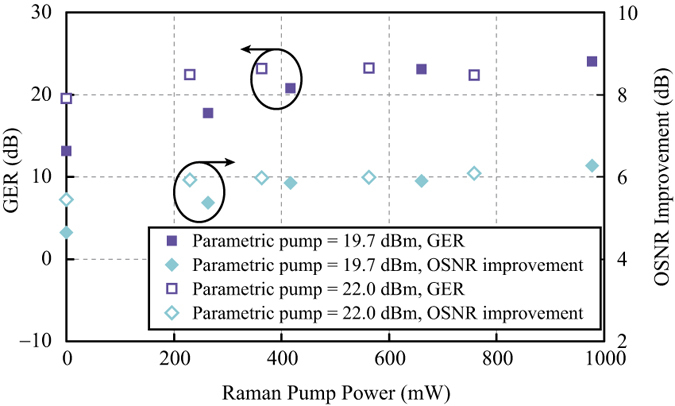
GER and OSNR improvement of Raman-enhanced PS-FOPA. The Raman pump power was varied while the parametric pump power was fixed at 19.7 dBm (solid symbols) and 22.0 dBm (hollow symbols), respectively.

The measurements shown in Figs 4 and 5 were repeated at higher parametric pump powers of 19.7 dBm and 22.0 dBm, respectively. The maximum and minimum PS gains are indicated by pink and blue triangles respectively in Fig. 6. The solid symbols were used for parametric pump power of 19.7 dBm and the hollow symbols were used for 22.0 dBm pump power. The dashed grey lines were again results obtained from numerical calculation. Likewise, the net Raman gains experienced by the parametric pump at different Raman pump powers are plotted as circles in Fig. 6. The GER and OSNR improvement are represented by purple squares and cyan diamonds respectively in Fig. 7. It is observed that the improvement of GER saturates at high Raman pump powers. The observed saturation is attributed to the limited OSNRs of the input signal and idler. As a consequence, the measurement of the minimum PS gain was eventually hindered by the PI amplification of the noise. Hence, the measured GER no longer increased with the Raman pump power. More detailed explanation on how the input OSNR affects the saturation of the GER can be found in the Method section.

The slight discrepancy between the experimental and simulation results is explained as follows. At large Raman pump powers, the simulated maximum gains are smaller than the experimental values as shown in Figs 4 and 6. The differences are attributed to underestimation of the idler power in the calculation. This is evidenced by the >6 dB OSNR improvement shown in Fig. 7 as the Raman pump power increases^9^. From Equation (2), the PS gain can be higher than the PI gain by >6 dB when the idler power is stronger than the signal power. Therefore, we believe that this is the case at the input. The origin of the underestimated minimum PS gains as shown in Figs 4 and 6 is more complicated. First, there is an overestimation of the signal and idler OSNRs caused by the OSA based measurements, which underestimate the in-band noise. Unlike the out-of-band noise, the in-band noise cannot be removed by the optical processor. Therefore, it is subsequently amplified by EDFA2 and is stronger than the out-of-band noise at the input of HNL-DSF2. The overestimation in OSNR results in underestimation of the simulated minimum PS gain obtained from Equation (10), especially at large *G*_RA-PI_ values. Second, the unpolarized nature of the Raman pump results in amplified spontaneous emission (ASE) noise in all polarizations, including the one perpendicular to the polarization of the parametric pump, signal, and idler. The contribution of the ASE noise to the measurement of the minimum PS gain becomes significant at large Raman pump powers. Third, pump power fluctuations caused by cascaded amplification in the EDFAs, as well as parametric gain fluctuation resulted from phase modulation of the pump for SBS suppression, also affect the measurement accuracy in the case of minimum PS gain. Last, the amplitude and phase noises introduced by the optical processor unavoidably lead to higher measured values of minimum PS gains.

## Discussion

The experimental demonstration in this work relied on inline PSA by cascading a PI-FOPA and a (Raman-enhanced) PS-FOPA. The OSNRs of the input signal and idler were limited by loaded noise. The loaded noise originated from the residual ASE in the PI-FOPA, which was not completely rejected at the optical processer. The noise was amplified by EDFA2 prior to the PSA stage. Due to the relatively large loaded noise, the signal OSNR cannot be guaranteed for coherent detection of phase-encoded data based on our current resources. Nonetheless, Raman-enhanced processing is proved to increase the GER of a PS-FOPA in the presence of the loaded noise. Compared to the PI counterpart, the Raman-enhanced PS-FOPA improves the OSNR of the output signal. By introducing a feedback control loop^4^,^7^, the signal and idler can propagate in a separate path so that only the parametric pump undergoes amplification in EDFA2. With this modification of the setup, Raman-enhanced PS-FOPA can be applied for low-noise amplification and regeneration of phase-encoded data.

In addition, Raman enhancement is particularly useful in optimizing the GER of the conventional PS-FOPA when the parametric pump itself cannot provide sufficient power. Since the gain bands of both parametric and Raman amplifications can be tuned by the pump wavelengths, this scheme offers the potential of migrating to spectral regions where high power amplifiers like EDFAs are not available. By properly designing the zero dispersion wavelength of the HNLF^18^, PS-FOPA can still be facilitated.

In this work, a HNLF with negligible fibre attenuation (0.79 dB/km) was used. At high parametric pump powers, phase matching can be maintained along the fibre as the parametric pump is almost undepleted. Given sufficient pump power, high PI parametric gain *G*_PI_ and large GER can be achieved. In such a case, backward Raman amplification may not lead to a significant improvement in the GER. However, this low loss feature is not always true for other types of HNLFs, especially in the cases when novel materials or dopants are introduced in the fibre design. The bismuth oxide based HNLF, offering a large nonlinear coefficient of 1360/W/km, suffers from a huge attenuation coefficient of 0.8 dB/m^19^. The aluminium-doped HNLF, designed to achieve an enhanced SBS threshold, also has a relatively large attenuation coefficient of 15 dB/km^20^. In such HNLFs, Raman enhancement through backward pumping can compensate the power drop along the fibre and greatly improve the performance of PS-FOPA.

## Methods

### SBS suppression

The pump laser was phase-modulated by a three-tone (112 MHz, 558 MHz, and 1906 MHz) radio frequency (RF) signal to suppress SBS in the HNLFs. The three RF tones were generated from three voltage controlled oscillators. They were amplified by an electrical amplifier to 15.2 dBm, 15.7 dBm, and 16.2 dBm, respectively, to drive an electro-optical phase modulator with a 3 dB RF bandwidth of 10 GHz. The powers of the RF tones were adjusted by three voltage variable attenuators to minimize the power of the backscattered Stokes wave in HNL-DSF2 at an input parametric pump power of 24.0 dBm. Compared to the case when no phase-modulation was applied, the SBS threshold in HNL-DSF2 was increased by ~14 dB.

### PS-FOPA and Raman-enhanced PS-FOPA set-up

A tunable external cavity laser with 13 dBm CW output power, −145 dB/Hz relative-intensity noise, and 100 kHz linewidth, was used as the parametric pump. The emitted wavelength was fixed at 1553.5 nm. The 2-km HNL-DSF1 was characterized by measuring the gain shape of the PI-FOPA. The average zero dispersion wavelength of the fibre was estimated to be ~1545 nm. With an input pump power of 22.5 dBm, PI parametric amplification in HNL-DSF1 provided 13.9 dB on-off gain to the signal at 1549.5 nm, accompanied by the generation of an idler at 1557.6 nm. At the output of HNL-DSF1, the total power of the optical waves was attenuated to 19.0 dBm by a variable optical attenuator (VOA) before the optical processor. The optical processor was able to impose multi-passband filtering across the C-Band with precise control of the filter phase and amplitude. It was used to adjust the phases of the parametric pump, signal, and idler. Simultaneously, it introduced extra attenuation to the signal and idler to prevent gain saturation in the following PS-FOPA. The optical processor also filtered the out-of-band ASE noise generated in the PI-FOPA and pre-compensated the unequal signal and idler gains in EDFA2. The input parametric pump power to EDFA2 was fixed at 13.0 dBm. Since the change of EDFA gain would affect the OSNRs of the pump, signal, and idler, the output power of EDFA2 was fixed at 23.5 dBm. VOA2 was used to control the parametric pump power while maintaining nearly unchanged OSNRs for the pump, signal, and idler. The input signal OSNR was ~15.6 dB. The 1 km HNL-DSF2 used for the PS-FOPA and Raman-enhanced PS-FOPA had a nonlinear coefficient of 11.7/W/km and an attenuation coefficient of 0.79 dB/km. The dispersion coefficient and dispersion slope at 1550 nm were 0.02 ps/nm/km and 0.019 ps/nm^2^/km, respectively. The total PMD was 0.06 ps. The Raman pump was a fibre laser delivering unpolarized output at a central wavelength of 1455 nm. The linewidth of the Raman pump laser was close to 2 nm. Under backward pumping, the Raman gain peak wavelength and gain coefficient in HNL-DSF2 were measured to be 1555 nm and 3.8/W/km^14^. The residual Raman pump from the Raman-enhanced PS-FOPA was extracted by CIR2 to protect the upstream components and equipment. Port 3 of CIR2 was properly terminated to avoid back reflection. The resolution of the OSA was kept at 0.1 nm throughout the measurements.

### Simulation on PS-FOPA and Raman-enhanced PS-FOPA

The simulation of the PS-FOPA was based on solving the nonlinear Schrödinger equation (NLSE) using split-step Fourier method (SSFM)^21^. The equation was modified to incorporate the effect of Raman amplification. The modified NLSE becomes


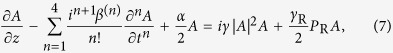


where *A* is the slowly varying envelope of the combined input waves, *β*^(*n*)^, *α, γ*, and *γ*_R_ are the *n*-th order propagation constant, fibre attenuation coefficient, nonlinear coefficient, and Raman gain coefficient of the HNLF, and *P*_R_ is the Raman pump power. To determine the evolution of *P*_R_ along the HNLF, the following two equations were applied


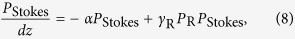



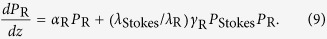


*P*_Stokes_ is the total power of the parametric pump, signal, and idler, if any, and *α*_R_ is the fibre attenuation coefficient at the Raman pump wavelength *λ*_R_. The parametric pump wavelength was used as the Stokes wavelength *λ*_Stokes_. The assumption of wavelength-independent Raman gain was valid because the wavelength differences among the parametric pump, signal, and idler were relatively small compared to the Raman gain bandwidth.

To compare the experimental and simulation results, Equations (7)–(9), 10, 10 were used to find the required Raman pump power to achieve certain signal gain *G*_R_*G*_RA-PI_ in a Raman-enhanced PI-FOPA. The corresponding direct Raman gain *G*_R_ was also obtained, and the Raman amplified PI parametric gain *G*_RA-PI_ was calculated.

Experimentally, the measurement of PI and PS gains described in the previous sections included the influence of the noise powers. The gain values were no longer governed simply by Equation (3) and (5), especially when we considered the minimum PS gains. In analysing the results that reflect the performance of PS amplification with a loaded-noise limited input, we took into consideration the input signal and idler OSNRs in calculating the maximum and minimum PS gains. By assuming identical signal and idler powers and OSNRs, the following equation was used


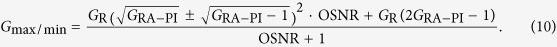


Here, OSNR is the input optical signal-to-noise ratio of the signal. For a conventional PS-FOPA, 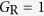 and *G*_RA-PI_ was reduced to *G*_PI_. From Eq. (10), the measured GER of the Raman-enhanced PS-FOPA is given by


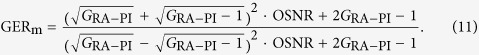


When G_RA-PI_ ≫ 1, GER_m_ will saturate at 2·OSNR+1.

## Additional Information

**How to cite this article**: Fu, X. *et al*. Raman-Enhanced Phase-Sensitive Fibre Optical Parametric Amplifier. *Sci. Rep.* **6**, 20180; doi: 10.1038/srep20180 (2016).
